# Kreativitätsförderung in der virtuellen Gruppenarbeit

**DOI:** 10.1365/s40702-021-00800-5

**Published:** 2021-09-28

**Authors:** Pia Gebbing, Xingyue Yang, Simon Michalke, Christoph Lattemann

**Affiliations:** Jacobs University Bremen gGmbH, Campus Ring 1, 28759 Bremen, Deutschland

**Keywords:** Kreativität, Virtuelle Zusammenarbeit, Virtuelle Teams, Digitales Lernen, Creativity, Virtual Collaboration, Virtual Teams, Digital Education

## Abstract

Laut OECD ist Kreativität eine der gefragtesten Soft Skills auf dem zukünftigen Arbeitsmarkt, was eine besondere Förderung in der Aus- und Weiterbildung notwendig macht. Problematisch ist, dass die zunehmende digitale Gestaltung von Lern- und Bildungsangeboten die Entwicklung von Kreativität im Lernprozess erschweren kann. Um zu untersuchen, welche Faktoren die Kreativität in der virtuellen Zusammenarbeit beeinflussen, wurde eine explorative Studie mit Studierenden eines fünftägigen online Design Thinking Workshops durchgeführt. Ziel ist es, aus Sicht der Lernenden die Faktoren zu identifizieren, die in der digitalen Lehre beachtet werden müssen, um kreative Gruppenarbeit zu ermöglichen. Die qualitative und quantitative Analyse der Bedürfnisse der Lernenden ergab drei Gruppen von Schlüsselfaktoren, die besonders bei der Gestaltung von Lehr- und Lernarrangements im digitalen Raum zu berücksichtigt sind: (1) Funktionalität und Eigenschaften der verwendeten technischen Tools, (2) psychologische Werte der Zusammenarbeit und Organisationskultur, (3) Motivation und Engagement.

## Abstract (en)

According to the OECD, creativity is one of the most sought-after soft skills on the future labor market, which is why creativity requires special attention in education and training. The problem is that the increasing use of digital formats in learning and education challenges the creative process. To investigate which factors influence creativity in virtual collaboration, an exploratory study was conducted with students in an online Design Thinking workshop spanning five days. The aim is to identify different factors, which affect creative work from the learners’ point of view. These insights help to design an appropriate digital teaching environment for creative group work. The qualitative and quantitative analysis of learners’ needs revealed three groups of design factors that need special attention in digital learning spaces: (1) functionality and characteristics of the technical tools, (2) psychological values of collaboration and organizational culture, (3) motivation and engagement.

## Einleitung: Kreativität als Schlüsselkompetenz in der digitalen Welt

Laut OECD gehört Kreativität zu den Schlüsselkompetenzen des 21. Jahrhunderts für Angestellte (Vincent-Lancrin et al. 2019). Während Lehrer und Bildungspolitiker Kreativität als wichtiges Lernziel ansehen, ist weitgehend unklar, was Kreativität in der Aus- und Weiterbildung ausmacht und wie sie zu fördern ist. Schon allein bei dem Verständnis von Kreativität gibt es oft verschiedene Ansätze. Zum einen können individuelle Merkmale, wie Persönlichkeitseigenschaften betrachtet werden (Person), man kann sich fragen, was genau eine kreative Idee ausmacht (Product), auf welchem Weg man ein kreatives Ergebnis erreicht (Process) und in welcher Umgebung Kreativität im besonderen Maßen gefördert wird (Press) (Rhodes 1961). Unserer Untersuchung liegt die Annahme zu Grunde, dass kreative Ideen zum einen anders, neu und innovativ sind, aber auch qualitativ hochwertig und angemessen für die anstehende Aufgabe sein müssen (Sternberg 2019). In diesem Beitrag beschäftigen wir uns vor allem mit Prozessen sowie mit Umgebungsvariablen, die Kreativität fördern.

Der Ausbruch der globalen Coronavirus-Pandemie (COVID-19) hat dazu geführt, dass der Unterricht an Bildungseinrichtungen über einen langen Zeitraum hinweg online stattfinden musste. Ebenso hat die Pandemie zu einer intensivierten virtuellen Kollaboration innerhalb und zwischen Unternehmen geführt. Die virtuelle Zusammenarbeit ist jedoch oft weniger effektiv als die Zusammenarbeit Vorort (Meluso et al. 2020). Da auch über die Krise hinaus die Digitalisierung der Aus- und Weiterbildung weiter im Fokus steht (Bundesministerium für Bildung und Forschung 2019), müssen bestehende Erkenntnisse zur Förderung von wichtigen Kompetenzen, wie der Kreativität, auf das digitale Format zugeschnitten und deren Wirksamkeit geprüft werden. Ziel dieses Beitrags ist, ein Verständnis dafür zu gewinnen, welche Faktoren für die Kreativitätsentwicklung in virtuellen Teams wichtig sind, um daraus Erkenntnisse zur Gestaltung von zukünftigen Lehr- und Lernarrangements abzuleiten. Um dieses Ziel zu erreichen, führen wir eine explorative Studie zur Vermittlung von Kreativität durch und befragen Mitglieder virtueller Teams, die einen digitalen Design Thinking Workshops absolviert haben. Wir verstehen virtuelle Teams als Gruppen von Menschen, die ein gemeinsames Ziel anstreben, über Standorte verteilt sind und durch den Einsatz von Informations- und Kommunikationstechnologie (IKT) in unterschiedlichem Ausmaß miteinander kommunizieren (Alahuhta et al. 2014).

## Theoretische Grundlagen zur Gestaltung von kreativen Umgebungen

Zwar gibt es viele Erkenntnisse zur Kreativitätsförderung in Bezug auf klassische, physische Lern- und Lehrsettings (Sternberg 2019), die Umstellung auf digitales Lehren und Lernen und die Arbeit in räumlich verteilten Teams, stellen den Lehrenden und Lernenden jedoch vor neue Herausforderungen. Verarmte Kommunikationskanäle beeinflussen die virtuelle Gruppenarbeit durch das Fehlen von spontanem, informellen Feedback durch Gestik und Körperhaltung, sowie asynchrone oder zeitlich verzögerte Kommunikation (Gilhooly et al. 2013). Auch neuere Studien zeigen, dass trotz Weiterentwicklung der Technologien, die virtuelle Umgebung hinderlich wirkt auf die Entwicklung von Empathie, Vertrauen, intentioneller Kommunikation und psychologischer Sicherheit, sowie den Umgang mit Diversität erschwert (Meluso et al. 2020) – dabei haben vorangehende Studien in physischen Teams gezeigt, dass gerade diese Faktoren einen positiven Einfluss auf Kreativität haben können (Amabile et al. 1996; Sternberg 2019).

Erste Anhaltspunkte zur Gestaltung einer kreativen, digitalen Lehr- und Lernumgebung bietet das Triple Path Framework (Dul 2019). Es wurde entwickelt für die Gestaltung kreativer physischer Umgebungen, wie Arbeits- und Büroräume. Demnach gibt es drei Schlüsselfaktoren einer physischen Umgebung, die zu mehr Kreativität anregen können: Funktionalität (Functionality), Bedeutung (Meaning) und Stimmung (Mood) (Dul 2019).

Funktionalität beschreibt die Art und Eigenschaften von Werkzeugen, Materialien und Gegenständen, die für die Durchführung kreativer Aktivitäten notwendig sind. In der physischen Arbeitsumgebung kann es sich hierbei zum Beispiel um ausreichend Tageslicht, und Materialien wie Stift und Papier handeln. Im digitalen Raum kommen die eingesetzten Technologien hinzu. Funktionale Arbeitsumgebungen sollten nach Dul (2019) instrumental, anpassungsfähig und frei von Ablenkungen sein.

Der erste Faktor in Dul’s Konzept, der das kreative Verhalten beeinflusst, Instrumentalität, lässt sich mit den Überlegungen des Technology Acceptance Models (TAM) von Davis (1989) operationalisieren: eine instrumentelle, digitale Arbeitsumgebung wird eher vom Nutzenden akzeptiert und steigert die wahrgenommene Benutzerfreundlichkeit (Perceived Ease of Use) und die wahrgenommene Nützlichkeit (Perceived Usefulness).

Als zweiten Faktor führt Dul (2019) die psychologische Bedeutung der Umgebung an. Nach kontextuellen Theorien zur organisationalen Kreativität ist es die psychologische Bedeutung (meaning) von Umweltereignissen, die kreatives Verhalten maßgeblich beeinflusst (Amabile et al. 1996). Die Umgebung muss also gewisse Werte und Bedeutungen widerspiegeln, die wichtig sind für die kreative Entfaltung. Nach Dul (2019) gehören dazu Freiheit, Inspiration, Interaktionsmöglichkeiten, Entspannung und Privatsphäre. Nach Amabile et al. (1996) lassen sich diese psychologischen Werte der Organisationskultur zuordnen. Die Organisationskultur bezieht sich auf die Mission, die Ziele, die Erwartungen und die Werte eines Unternehmens, bzw. eines Lehr‑/Lernarrangements, an denen sich die Mitarbeiter, bzw. Lernenden orientieren. Laut Amabile et al. (1996) gibt es acht relevante Einflussfaktoren auf die Kreativität in der Organisationskultur: (1) herausfordernde Gestaltung der Arbeit, (2) Freiheit bei der Ausführung der Aufgaben, (3) Unterstützung von Kreativität durch die Organisation und (4) den direkten Vorgesetzten, (5) Unterstützung durch die Arbeitsgruppe, sowie (6) organisatorische Hindernisse, (7) Vorhandensein der notwendigen Ressourcen und (8) Arbeitsdruck. Ein weiterer Faktor, der oft mit erfolgreicher Teamarbeit in Verbindung gebracht wird, ist die psychologische Sicherheit – das Gefühl sich frei ausdrücken und Kritik äußern zu können, ohne Angst vor negativen Folgen für das Selbstverständnis, den Status oder die Arbeit zu haben (Kahn 1990).

Der dritte Faktor in Dul’s Konzept, der das kreative Verhalten beeinflusst, Stimmung und Affekt, besagt, dass eine Umgebung uns in eine bestimmte positiv oder negativ aktivierende Grundstimmung versetzt, was sich wiederum auf die Kreativität auswirkt (Dul 2019). Die Ergebnisse einer Metaanalyse haben gezeigt, dass die Gestaltung der Umgebung einen signifikanten Einfluss darauf haben kann, wie wir uns fühlen, und dass eine positiv aktivierende Stimmung Kreativität fördert (Baas et al. 2008). Gleichzeitig scheint es, dass die intensive Nutzung von IKT eine zusätzliche kognitive Belastung darstellt und für ein erhöhtes Stressniveau sorgt – auch als Technostress bezeichnet (Ayyagari et al. 2011; Gerdiken et al. 2021). Dies könnte dazu führen, dass die kreative Leistung der Gruppenmitglieder vermindert wird.

Die meisten der existierenden Theorien zur Kreativitätsförderung richten sich nicht auf eine Anwendung im digitalen Umfeld, sondern beziehen sich eher auf die physische Arbeitsumgebung. Früherer Forschungsergebnisse zeigen jedoch, dass sich die Zusammenarbeit im digitalen Raum deutlich von der im direkten Kontakt unterscheidet. Nur wenige Studien haben Kreativität in der digitalen Zusammenarbeit erforscht und auch das Triple Path Framework von Dul (2019) wurde bisher nicht in der digitalen Umgebung getestet. Hier ergibt sich folglich eine Forschungslücke, die wir mit der vorliegenden Untersuchung adressieren. Im Hinblick auf die zunehmende Zahl digitaler Lernangebote haben wir untersucht, welche Umgebungsfaktoren für die Förderung der Kreativität in der digitalen Hochschulbildung relevant sind.

Das experimentelle Setting für diese Forschung bildet ein fünftägiger online Design Thinking (DT) Workshop. DT ist ein bewährter Innovationsansatz, der es ermöglicht, kreative und innovative Lösungen für (komplexe) Probleme zu entwickeln, die sich an den Bedürfnissen der Nutzer orientieren (Lattemann et al. 2017). Der DT Prozess wechselt zwischen Phasen des divergenten und konvergenten Denkens, zwei häufig verwendete Konzepte der Kreativitätsforschung (Sternberg 2019). Der Ansatz, eine konkrete Lösung für ein Problem zu finden, wird als konvergentes Denken bezeichnet. Divergentes Denken ist der Prozess des Denkens, der vielfältige mögliche Lösungen ermittelt, um kreative Ideen zu generieren. Frühere Untersuchungen zu Arbeiten in Online-Settings haben zudem gezeigt, dass semi-virtuelle und virtuelle DT Workshops zu einem hohen Maß an gemeinsamem Verständnis, Zufriedenheit und wahrgenommener Effektivität führen können, und dass kreative Lösungen in kürzerer Zeit als in nicht-digitalen Umgebungen erarbeiten werden können (Lattemann et al. 2017).

## Methoden

Um zu untersuchen, welche Faktoren die Kreativität in einer virtuellen Zusammenarbeit beeinflussen, wurde eine explorative Umfrage mit 31 Studierenden an einer deutschen Universität mit internationaler Studierendenschaft durchgeführt. Der Workshop fand an fünf aufeinander folgenden Tagen ganztägig (8 h) statt, was es den Studierenden erlaubte, sich intensiv mit den eingesetzten Methoden und Technologien vertraut zu machen. Der Workshop wurde von geschulten DT Trainern begleitet, die mit Technologien zur virtuellen Zusammenarbeit erfahren sind. Die Teilnehmer des DT Workshops bildeten fünf Gruppen à sechs Personen. Die Zuteilung zu den Gruppen erfolgte systematisch unter Berücksichtigung von Unterschieden in studienbezogenen Bereichen mit dem Ziel, möglichst heterogene Teams zu schaffen. Jede Gruppe bearbeitete eine reale, von einem Unternehmen gestellte Fragestellung. Der DT Ansatz bildet die Rahmenstruktur, um eine Problemstellung zu bearbeiten. Entsprechend des Ablaufs des DT Prozesses kamen sowohl Methoden zum Einsatz, die das divergente Denken fördern (z. B. Interviews, Brainstorming Methoden) oder konvergentes Denken unterstützen (z. B. durch die Erstellung eines Prototyps). Kreativmethoden unterstützen den Arbeitsprozess der Gruppe auf dem digitalen Whiteboard, z. B. durch Templates und Vorlagen.

Die Studierenden nutzten eines der beiden digitalen Whiteboards Mural oder Miro, um Unterschiede in der Funktionalität zu vergleichen. Beide Whiteboards ähneln sich in der Funktionsweise, unterscheiden sich jedoch in Design und Handhabung. Miro und Mural sind Online-Kollaborationsplattformen, die verteilten Teams verschiedene Funktionalitäten zu Verfügung stellen um effektiv zusammenzuarbeiten. Einem physischen Whiteboard nachempfunden, kann auf dem Whiteboard mit digitalen Haftzetteln, Stiften oder auch Bildern gearbeitet werden. Die Ähnlichkeit zu einem physischen Whiteboard soll die Nutzerfreundlichkeit steigern und den Gebrauch intuitiver machen. Die Gruppenmitglieder können zeitgleich auf dem Board zusammenarbeiten, einander folgen oder auch Abstimmungen erstellen. Für die verbale Teamkommunikation wurde Microsoft Teams (MS Teams) zur Verfügung gestellt. Zusätzlich konnten die Teilnehmer weitere IKT nach eigenem Bedarf nutzen und wurden explizit durch den Moderator dazu ermutigt, von dieser Flexibilität Gebrauch zu machen.

Nach Ende des Workshops wurde zeitgleich mit der Kursevaluation eine Umfrage durchgeführt, die den Einfluss virtueller Settings auf die Kreativität untersucht. Hierzu wurde ein Fragebogen mit insgesamt 29 Fragen erstellt, die sich aus vorangehenden Forschungen ableiteten (Amabile et al. 1996; Davis 1989; Rhodes 1961; Baas et al. 2008; Sternberg 2019). Es wurden die folgenden sechs Aspekte erfasst: (1) Allgemeine Auswirkung des virtuellen Settings auf die Gruppenleistung und persönliche Leistung; (2) Wahrgenommener Nutzen und wahrgenommene Benutzerfreundlichkeit; (3) Auswirkung des virtuellen Settings auf die Kreativität; (4) Auswirkung des virtuellen Settings auf die divergenten und konvergenten Phasen im DT; (5) Allgemeine Evaluation des Kurses, sowie (6) Einfluss des virtuellen Settings auf die Stimmung. Der Fragebogen enthielt 22 geschlossene Fragen auf einer 5‑Punkte-Likert-Skala (1 = sehr negativ/stimme überhaupt nicht zu, 3 = neutral, 5 = sehr positive/stimme voll umfänglich zu), aufgelistet in Tab. 1.

**Tab. 1:** Antworten zu den geschlossen Fragen

Nr	Geschlossene Fragen	*n*	Median	Mean (M)	Standard Deviation (SD)
F1	How would you describe the impact of the virtual setting on your GROUP’s performance?	23	4	3,67	1,11
F2	How would you describe the impact of the virtual setting on your PERSONAL performance?	23	4	3,90	1,12
F3	How did the online setting influence your mood/the way you feel?	23	3	3,52	1,03
F4	The virtual tools were useful for our group work	23	5	4,38	1,02
F5	Using virtual tools enhances the effectiveness of working creatively	23	5	4,33	0,86
F6	The virtual tools we used are easy and understandable	23	5	4,19	1,12
F7	By using virtual tools, my group members and I can cooperate easily	23	4	4,00	1,18
F8	Interaction with the group members online was uncomplicated	23	4	3,81	1,40
F9	I found it easy to get the tools to do what I want	23	4	3,95	1,02
F10	I found the virtual tools intuitive	23	4	4,05	1,02
F11	I intend to revisit/reuse the tools in the future	23	5	3,95	1,43
F12	Using virtual tools improves my creative performance compared to working in a physical classroom environment (e.g. using post-its on a whiteboard)	23	4	3,52	1,17
F13	Did the virtual setting in general help or hinder your creativity?	23	4	3,76	0,94
F14	I think that it is easy to foster students’ creativity in the context of face to face learning	23	4	3,81	0,93
F15	I think that it is easy to foster students’ creativity in the context of online learning	23	4	3,38	1,12

1 = sehr negativ/stimme überhaupt nicht zu, 3 = neutral, 5 = sehr positive/stimme voll umfänglich zu

**Tab. 2:** Von Studierenden genannte Faktoren, die die kreative Arbeit in virtuellen Gruppen beeinflussen

Einflussfaktoren auf Kreativität in virtuellen Gruppen					
Faktor	Q1	Q2	Q3	Total	% Aller Argumente
*Werte und Organisationskultur*	*33*	*20*	*32*	*85*	**55**
Interaktion und Kommunikation	10	5	5	20	**13**
Nachvollziehbarkeit der Gedanken	3	3	7	13	**8**
Effizienz der Aufgabenbearbeitung	4	1	3	8	**5**
Präsenz/Überprüfbarkeit des Einzelbeitrag	6	1	1	8	**5**
Simultanes Arbeiten	4	2	1	7	**5**
Freiheit und Autonomie	1	1	5	7	**5**
Konzentration	2	2	2	6	**4**
Inspiration	1	0	3	4	**3**
Multitasking	0	3	1	4	**3**
Ermutigung zur Kreativität	1	0	2	3	**2**
Psychologische Sicherheit	0	2	1	3	**2**
Privatsphäre	0	0	1	1	**1**
Druck (Zeit/Arbeitsaufwand)	1	0	0	1	**1**
*Funktionalität*	*21*	*11*	*19*	*51*	**33**
Generelle Funktion	11	5	4	20	**13**
Einfachheit der Bedienung	5	2	5	12	**8**
Anpassungsfähig	2	1	5	8	**5**
Frei von Ablenkungen	0	2	2	4	**3**
Wahrgenommener Nutzen	2	1	1	4	**3**
Zeit Effizienz	1	0	2	3	**2**
*Motivation und Stimmung*	*4*	*12*	*2*	*18*	**12**
Motivation und Engagement	3	5	1	9	6
Positive Aktivierung/Spaß	0	4	1	5	3
Entspannung/Techno-Stress	1	3	0	4	3

Q1 Einfluss auf das Gruppenergebnis, Q2 Einfluss auf individuelle Arbeitsergebnisse, Q3 Was hilft oder verhindert eine digitale Gruppenarbeit?

Drei offene Fragen gaben den Studierenden die Möglichkeit, sich ausführlicher zu ihren Erfahrungen zu äußern. Die drei Fragen lauteten Q1: „Können Sie Beispiele nennen, inwieweit Ihre Gruppenleistung durch das virtuelle Setting beeinflusst wurde?“, Q2: „Können Sie Beispiele nennen, inwieweit Ihre persönliche Leistung durch das virtuelle Setting beeinflusst wurde?“ und Q3: „In wie fern hat Ihnen das virtuelle Setting geholfen oder Sie daran gehindert, kreativ zu sein?“.

Die Analyse der durch offene Fragen erhoben qualitativen Daten erfolgt im Sinne der Grounded Theory (Flick 2016) durch systematische Kodierung. Alle Argumente wurden im ersten Schritt offen kodiert, d. h. die Antworten auf die offenen Fragen wurden in einzelne Sinneinheiten, oder auch Faktoren gegliedert, die verschiedene Einflüsse auf die Kreativität beschreiben. Insgesamt wurden von den 23 Studierenden 170 verschiedene einzelne Faktoren genannt, die sich zum Teil entsprachen und daher zu insgesamt 22 Kategorien zusammengefasst wurden. Die Kategorisierung wurde von zwei Beobachtern unabhängig voneinander durchgeführt und anschließend verglichen. Im nächsten Schritt wurden die von den Studierenden genannten Einflussfaktoren deduktiv, theoriegeleitet zu Überkategorien zusammengefasst. Dabei wurde Bezug genommen auf die eingangs erwähnten Theorien, wie das Triple Path Framework von Dul (2019), den Merkmalen organisatorischer Kulturen nach Amabile et al. (1996) und Aspekte des TAM (Davis 1989).

## Ergebnisse

Die Datenerhebung fand im Januar 2021 statt. Von den ursprünglich 31 angemeldeten Studierenden, füllten 23 den Evaluierungsfragebogen aus. Insgesamt wurden die Auswirkungen der Arbeit im virtuellen Setting auf die kreative Leistung von den Studierenden als neutral bis positiv bewertet (M = 3,76, SD = 0,9, Median = 4), dies galt sowohl für die Gruppenleistung (M = 3,67, SD = 1,1, Median = 4) als auch die persönliche Leistung (M = 3,9, SD = 1,1, Median = 4). Auf die Frage, in welchem Format ein solcher DT Workshop in Zukunft angeboten werden solle, antworteten 17 Studierende mit Hybrid-Format, fünf Studierende bevorzugten den Präsenzunterricht und drei Studierende bevorzugten ein Online-Format (mehrfach Nennung waren möglich).

Welche Faktoren die kreative Gruppenarbeit beeinflussen (Tab. 2), wurde durch die Analyse der offenen Antworten mittels Kodierung festgestellt. Die Ergebnisse lassen sich zu den drei Kategorien zusammenfassen: (1) Technische Funktionalität, (2) Werte der Arbeits- und Organisationskultur und die (3) Motivation und Stimmung der Lernenden.

### Funktionalität

Ein Drittel (33 %) der genannten Einflussfaktoren beschreiben wie die Funktionalität der eingesetzten technischen Hilfsmittel (z. B. digitale Whiteboards, WhatsApp) sich auf die kreative Leistung auswirkten.

Während der Durchführung des einwöchigen DT Workshops gab es einen unerwarteten Internetausfall über einen Zeitraum von 23 h, während dessen die Studierenden nur eingeschränkt Zugriff auf die digitalen Inhalte und Plattformen hatten. Einen deutlich negativen Einfluss auf die Zusammenarbeit hatten daher technische Probleme („Aufgrund eines Netzwerkausfalls war eine konstante, klare Kommunikation nicht möglich“). 30 % der Studierenden sagten, dass sie sich durch die technischen Probleme beeinträchtigt fühlten.

Tendenziell war die Bewertung der Funktionalität der eingesetzten technischen Hilfsmittel positiv: „Die virtuelle Umgebung hat die Kreativität gefördert, denn durch die Nutzung der Plattformen konnte ich persönlich meine Gedanken leichter und auf unterhaltsame Art darstellen, was wiederum dazu führte, dass ich leichter Verbindungen herstellen konnte. Durch die Nutzung der verschiedenen Funktionen von Miro war es für uns alle einfacher, über die Herausforderung und mögliche Lösungen nachzudenken, da wir alle im selben Dokument arbeiteten.“. Es konnte kein signifikanter Unterschied zwischen den beiden Whiteboards Miro und Mural festgestellt werden. Die digitalen Werkzeuge wurden als relativ einfach und verständlich (M = 4,19, SD = 1,1, Median = 5) und in der Anwendung als leicht (M = 3,95, SD = 1,0, Median = 4), intuitiv (M = 4,05, SD = 1,0, Median = 4) und unkompliziert (M = 3,81, SD = 1,4, Median = 4) wahrgenommen. Die Werkzeuge waren nützlich (M = 4,38, SD = 1,0, Median = 5) und effektivitätssteigernd (M = 4,33, SD = 0,9, Median = 5). Insgesamt gaben die Studierenden an, dass sie keinen signifikanten Unterschied zwischen der Benutzerfreundlichkeit von virtuellen und physischen Whiteboards wahrnehmen (M = 3,52, SD = 1,2, Median = 4).

### Werte und Organisationskultur

55 % der von den Studierenden genannten Faktoren beziehen sich auf die Werte und Normen der Zusammenarbeit in der Gruppe, 15 % auf Werte der Organisationskultur.

Die Analyse der offenen Fragen ergab, dass Interaktion und Kommunikation einen maßgeblichen Einfluss auf den subjektiven Erfolg der kreativen Gruppenarbeit hatten. Mehrfach positiv hervorgehoben wurde, dass Ideen durch die Nutzung der Whiteboards leichter visualisiert und Einzelbeiträge besser nachvollzogen werden können. Auch dass alle Gruppenmitglieder durch das digitale Whiteboard gleichzeitig zusammenarbeiten und die Synchronität dadurch deutlich erhöht wurde, wurde oft positiv erwähnt. Nach Aussage der Befragten verbessere eine effiziente Zusammenarbeit das kreative Ergebnis der Gruppe.

Zu den positiven Werten der Organisationskultur gehören die Freiheit sich kreativ auszudrücken, Autonomie, und eigenverantwortliches Arbeiten und die selbstständige Arbeits- und Zeiteinteilung. Die digitale Arbeitsumgebung wurde von vielen Studierenden als konzentrationsförderlich, individuell anpassungsfähig und inspirierend wahrgenommen. Hinderlich wirke sich dagegen Druck durch Zeit- und Arbeitsbelastung auf die Kreativität aus.

Präsenz, Anonymität und Privatsphäre im digitalen Setting wurden von den Studierenden sehr unterschiedlich wahrgenommen. Einerseits war es wichtig, dass der Einzelbeitrag der Teammitglieder nachvollziehbar und überprüfbar ist: „Es war inspirierend, andere gleichzeitig arbeiten zu sehen, was die Zusammenarbeit unter uns verstärkte.“. Andererseits wurden Rückzugsmöglichkeiten und Privatsphäre als besonders wertvoll erachtet: „Ich brauche Stille und Zeit für mich zum Nachdenken. In einer Offline-Umgebung muss ich mich vielleicht von anderen Teamkollegen abkapseln, wodurch mir vielleicht Inspirationen entgehen.“ Ein Student fühlte sich im Online-Setting beobachtet und unter Druck gesetzt: „Ich mag es nicht, während des Brainstormings und der Gruppenarbeit ständig beobachtet zu werden. Die Möglichkeit, jede Änderung, die Teilnehmer/Lehrer an Miro vornehmen, zu sehen, hat mich ehrlich gesagt sehr gestresst, weil ich das Gefühl hatte, der Gruppenarbeit zuliebe etwas hinzufügen zu müssen, was aber den Denk‑/Lernprozess des Kurses selbst reduziert und untergraben hat.“. Der Wunsch nach einerseits mehr Anonymität und Privatsphäre auf der einen und Nachvollziehbarkeit der Einzelbeiträge („Accountability“) und regem Austausch auf der anderen Seite verursachte gelegentlich Spannungen in der Zusammenarbeit.

### Motivation und Stimmung

Das Triple Path Model von Dul (2019) besagt, dass die Stimmung, in die eine Umgebung den Menschen versetzt, einen direkten Einfluss auf die Kreativität des Menschen hat. In unserer Umfrage wurde der Einfluss des digitalen Settings auf die Stimmung als neutral bewertet (M = 3,52, SD = 1,03, Median = 3). In der Analyse der offenen Fragen zeigte sich jedoch, dass insgesamt 12 % der Antworten sich auf Motivations- und Stressfaktoren bezogen. Zu den positiven Faktoren gehörten die persönliche Motivation und das wahrgenommene Engagement der Gruppenmitglieder. Negative Einflussfaktoren bezogen sich auf wahrgenommenen Stress, ausgelöst durch das digitale Setting: „Wir mussten mehrere Stunden lang auf einen Bildschirm schauen, so dass wir nach dem Unterricht lange Pausen machten, bevor wir wieder mit der Arbeit begannen“. Von den Studierenden wurden drei Auslöser für Stress genannt: (1) Probleme mit der Internetverbindung und damit die Unterbrechung der Interaktionsmöglichkeiten, (2) Ermüdungserscheinungen durch lange Bildschirmarbeit und (3) die doppelte kognitive Belastung, da sie zum einen lernen mussten, mit einer (neuen) Technologie umzugehen, zum anderen die gestellte Aufgabe bearbeiten mussten.

## Interpretation und Einordnung der Ergebnisse im Kontext bisheriger Forschung

Ausgehend von der Frage, welche Faktoren für die Förderung von Kreativität in der digitalen Lern- und Lehrumgebung relevant sind, werden im Folgenden die Ergebnisse in den theoretischen Kontext eingebettet und mit den Erkenntnissen der Kreativitätsforschung in der digitalen Bildung in Beziehung gesetzt. Bei der Interpretation der Ergebnisse wird ein nutzerzentrierter Ansatz verfolgt, d. h. es wird besonders darauf geachtet, was aus der Perspektive des Lernenden wichtig ist.

### Funktionalität: Die richtigen digitalen Werkzeuge sind wichtig, aber nicht ausreichend

Die Funktionalität der eingesetzten IKT ist grundlegend, um Kreativität in der virtuellen Gruppenarbeit zu ermöglichen (Dul 2019). Nach unserer Erhebung und unter Bezugnahme auf die bestehende Literatur (Davis 1989; Redlich et al. 2017; Dul 2019) ergeben sich sechs kreativitätsfördernde Aspekte der IKT: Visualisierung, Synchronizität, Anpassungsfähigkeit, Reichhaltigkeit, wahrgenommene Einfachheit der Nutzung und wahrgenommene Nützlichkeit. Whiteboards wie Mural und Miro ermöglichen es, gleichzeitig an einem Thema zu arbeiten, Ideen zu notieren und Beiträge zu ordnen. Durch Visualisierung der Einzelbeiträge werden die Gedankengänge der Gruppenmitglieder verdeutlicht und für andere nachvollziehbar. Unsere Analyse zeigt, dass ein Angebot bereits existierender Vorlagen hilfreich ist, um den Arbeitsablauf zu strukturieren. Eine fachliche und didaktische Schulung in der Auswahl und dem sicheren Umgang mit den Technologien vor Beginn der inhaltlichen Arbeit ist notwendig, damit alle Gruppenmitglieder ihr kreatives Potenzial voll entfalten können, und um eine kognitive Doppelbelastung durch Erlernen der technischen Plattform und der inhaltlichen Arbeit zu vermeiden.

Die Ergebnisse unserer explorativen Befragung bestätigen, dass eine gut gestaltete IKT notwendig, aber nicht ausreichend ist, um Kreativität zu fördern. Es ist kaum verwunderlich, dass technische Probleme und eine instabile Internetverbindung zu den größten Störfaktoren für eine kreative und unterstützende Arbeitsumgebung in einem virtuellen Raum gehören. Interessanterweise fiel die kreative Arbeit durch die Studierenden trotz des 23 stündigen Internetausfalls positiv aus, was darauf hindeutet, dass es weitere Faktoren gibt, die mangelnde Funktionalität in gewissem Maße kompensieren können.

### Werte: Freiheit, Autonomie und Zusammenarbeit in der kreativen Gruppenarbeit

Unsere qualitative Analyse zeigte, dass technische Probleme durch die Gruppenmitglieder kompensiert werden, wenn eine hohe Motivation und Leistungsbereitschaft besteht. Erfolgreiche kreative Gruppenleistungen erfordern dafür eine Arbeits- und Organisationskultur, die bestimmte Werte sowie ein hohes Maß an Motivation, Leistungsbereitschaft und Engagement voraussetzt und fördert. Die von den Studierenden in unserer Umfrage genannten Werte decken sich mit Konzepten, die in bestehender Literatur zur Kreativität in physischen Umgebungen diskutiert wurden (Dul 2019; und Amabile et al. 1996). Wichtig ist, dass die Gruppenmitglieder explizit ermutigt werden, kreativ zu sein, bei gleichzeitiger Förderung von Autonomie und Freiheit im Aufgaben- und Zeitmanagement (Amabile 1983). Hierdurch schafft jede Gruppe eigene Werte, die die Zusammenarbeit, Kommunikation und Aufgabenbearbeitung steuern.

Zugleich beobachten wir im digitalen Raum ein Spannungsverhältnis zwischen Präsenz und Anonymität – also zwischen dem Bedürfnis nach regem Austausch im Sinne von „sehen und gesehen werden“ und Rückzug in die eigene Privatsphäre. Ein Gefühl der Präsenz wird durch das zeitgleiche Arbeiten auf dem digitalen Whiteboard geschaffen und ermöglicht den Einzelbeitrag der anderen visuell nachzuvollziehen.

Zeitgleich ermöglicht das Arbeiten in räumlich getrennten Teams Rückzugsmöglichkeiten zur Reflexion und Einzelarbeit. Eine Studie zur Zoom-Fatigue (Bailenson 2021) stellte fest, dass Teilnehmer im digitalen Setting sich zum Teil gehemmter und stärker beobachtet fühlen, ein stärkeres Bedürfnis nach Privatsphäre haben und befürchten, durch Videoübertragung zu viel von sich Preis zu geben. Anonymität und Privatsphäre durch Momente, in denen man sich unbeobachtet fühlt, können dazu führen, dass neue Ideen „gewagt“ werden, ohne dass andere diese gleich bewerten können. Wichtig ist hierbei einen Rahmen zu schaffen, in dem die Teilnehmenden sich sicher fühlen und einander vertrauen. Studien zu Brainwriting haben gezeigt, dass in Phasen der Ideengenerierung die Anzahl neuer Ideen deutlich steigt, wenn jeder erst für sich nachdenkt und seine Gedanken nicht von den Argumenten des Vorredners beeinflusst werden (Sternberg 2019).

Für die erfolgreiche Bearbeitung einer kreativen Problemstellung in einer Gruppe reicht es jedoch nicht, wenn einzelne Gruppenmitglieder im Schutze ihrer Anonymität innovative Ideen generieren. Sie müssen auch in der Gruppe zusammengebracht, besprochen und koordiniert werden. Wir beobachten, dass es wichtig ist, die Einzelbeiträge nachvollziehen zu können und so sicherzugehen, dass jedes Gruppenmitglied einen angemessenen Beitrag leistet. Hilfreich ist daher insbesondere der Einsatz von digitaler Whiteboards, die es erlauben, Ideen zu visualisieren, zu besprechen und zu koordinieren und darüber abzustimmen.

Insgesamt gab es große individuelle Unterschiede in der Präferenz für eine offene bzw. anonyme Zusammenarbeit. Es ergibt sich demnach ein Spannungsfeld im digitalen Raum zwischen dem Bedürfnis nach Anonymität und Präsenz, das von dem Studieren individuell sehr unterschiedlich wahrgenommen wird. In unserer Analyse empfand die Mehrheit der Studierenden das Gefühl der Präsenz als positiv und motivierend. Vereinzelt fühlten sich Studierende jedoch auch beobachtet und unter Druck gesetzt. Andere hatten das Gefühl, stillere, bzw. anonym arbeitende Gruppenmitglieder aus den Augen zu verlieren. Es kann daher helfen, Phasen der Anonymität und verstärkten Präsenz bewusst in der Gestaltung von digitalen Formaten einzusetzen, um vielfältigere Ideen zu generieren und individuellen Bedürfnissen zu entsprechen.

### Motivation und Techno-Stress: Das Zünglein an der Waage

Dass vor allem eine positive, aktivierende Stimmung sich förderlich auf Kreativität auswirkt, konnten Baas et al. (2008) in einer Metaanalyse bestätigen. Den von Dul (2019) beschriebenen Einfluss der Arbeitsumgebung auf die Stimmung und damit auf die Kreativität konnten wir in unserer Studie jedoch nicht nachweisen. Trotz massiver technischer Probleme während der Durchführung der Studie gaben die Studierenden an, dass Ihre Stimmung neutral bis positiv blieb. Die Studierenden schrieben den Erfolg ihrer Arbeit vor allem der hohen Arbeitsmotivation und Anstrengung der Teammitglieder zu und konnten so technische Probleme kompensieren.

Die Studierenden berichteten von wahrgenommenem Stress, ausgelöst durch das digitale Setting. Die genannten Stressoren, wie lange Bildschirmzeit und instabile Kommunikation, beziehen sich konkret auf die digitale Zusammenarbeit und sind typische Anzeichen von Technostress (Gerdiken et al. 2021). Diese Störquellen stellen eine zusätzliche Belastung dar, die Anspannung und Ablenkung erzeugt und sich negativ auf die Kreativität und Innovationsfähigkeit auswirken kann (Ayyagari et al. 2011; Gerdiken et al. 2021). Da Entspannung wichtig für den kreativen Ideenfluss ist (Dul 2019), sollte bei der kreativen Teamarbeit auf Anzeichen von Ermüdung reagiert und Technostress vermieden werden. Die Studienteilnehmer schätzten es insbesondere, selbstständig über Pausenzeiten zu bestimmen und flexibel zwischen Einzel- und Gruppenarbeit zu wechseln.

## Ausblick

Die Digitalisierung verändert Arbeitsplätze und Arbeitsweisen. Diese Entwicklung wurde durch die die COVID-19-Pandemie nochmals forciert. Diese Entwicklungen haben Einfluss auf die notwendigen Kompetenzen von Arbeitnehmern in der Zukunft. So zählt laut OECD Kreativität zu den gefragtesten Soft-Skills auf dem zukünftigen Arbeitsmarkt (Vincent-Lancrin et al. 2019). Die Förderung von Kreativität muss daher zukünftig in Bildungseinrichtungen eine größere Rolle spielen. Doch nicht nur die Anforderungen an die zu vermittelnden berufsbezogenen Kompetenzen verändern sich, auch das Format der Aus- und Weiterbildung wird zunehmend digitaler. Das Problem ist, dass die zunehmende digitale Gestaltung von Lern- und Weiterbildungsangeboten den kreativen Lernprozess erschweren kann. Der vorliegende Beitrag befasste sich daher mit Möglichkeiten der Förderung von Kreativität und Hindernissen der Entstehung von Kreativität in virtuellen Arbeitsumgebungen. Die vorgestellte explorative Studie zeigt drei Faktoren auf, die die Kreativität in der virtuellen Zusammenarbeit maßgeblich beeinflussen: (1) Technische Funktionalitäten der genutzten IKT, (2) Werte der Arbeits- und Organisationskultur und die (3) Motivation und Stimmung der Lernenden.

Unsere Studienergebnisse zeigen, dass die Möglichkeiten der Kommunikation und Interaktion sowie die Möglichkeiten der präsenten als auch der anonymen Kollaboration entscheidende Faktoren für eine erfolgreiche kreative Gruppenarbeit darstellen. Ebenso beeinflusst der vorgegebene Grad der Freiheit und Autonomie in der Gruppenarbeit die Kreativität, da diese Faktoren auf die Gruppendynamik und Gruppenprozesse wirken.

Unsere Analyse zeigt auf, dass es nicht ausreicht, bei der kreativen Arbeit im digitalen Raum nur an die Gestaltung der technologischen Plattform zu denken. Um Kreativität in einer digitalen Lehr- und Lernumgebung zu fördern, ist es wichtig, ein didaktisches Konzept zu erstellen, dass sich am Lernprozess orientiert und nicht ausschließlich am Endergebnis. Für die Gestaltung solcher didaktischen Konzepte zeigt unsere Analyse erste Ergebnisse auf. Es ist aber zu beachten, dass die Erkenntnisse durch größer angelegte Studien zu validieren zu generalisieren sind. Weitere Analysen sollten auf breitere Datenbasis gestellt werden und verschiedene Lehr- und Lernumwelten und Organisationsformate einbinden.
